# Identification of Specific Coronary Artery Disease Phenotypes Implicating Differential Pathophysiologies

**DOI:** 10.3389/fcvm.2022.778206

**Published:** 2022-03-10

**Authors:** Jona B. Krohn, Y Nhi Nguyen, Mohammadreza Akhavanpoor, Christian Erbel, Gabriele Domschke, Fabian Linden, Marcus E. Kleber, Graciela Delgado, Winfried März, Hugo A. Katus, Christian A. Gleissner

**Affiliations:** ^1^Department of Cardiology, Pulmonology and Angiology, University Hospital Heidelberg, Heidelberg, Germany; ^2^German Centre for Cardiovascular Research (DZHK) Partner Site Heidelberg/Mannheim, University of Heidelberg, Heidelberg, Germany; ^3^Department of Cardiology and Stroke Centre, Rottal-Inn Kliniken, Eggenfelden, Germany; ^4^Medical Clinic V, Medical Faculty Mannheim, University of Heidelberg, Mannheim, Germany; ^5^Clinical Institute of Medical and Chemical Laboratory Diagnostics, Medical University Graz, Graz, Austria; ^6^Synlab Academy, Synlab Holding Deutschland GmbH, Mannheim, Germany

**Keywords:** coronary artery disease, cardiovascular outcome, cardiovascular mortality, cardiovascular risk, coronary angiography

## Abstract

**Background and Aims:**

The roles of multiple risk factors of coronary artery disease (CAD) are well established. Commonly, CAD is considered as a single disease entity. We wish to examine whether coronary angiography allows to identify distinct CAD phenotypes associated with major risk factors and differences in prognosis.

**Methods:**

In a cohort of 4,344 patients undergoing coronary angiography at Heidelberg University Hospital between 2014 and 2016, cluster analysis of angiographic reports identified subgroups with similar patterns of spatial distribution of high-grade stenoses. Clusters were independently confirmed in 3,129 patients from the LURIC study.

**Results:**

Four clusters were identified: cluster one lacking critical stenoses comprised the highest percentage of women with the lowest cardiovascular risk. Patients in cluster two exhibiting high-grade stenosis of the proximal RCA had a high prevalence of the metabolic syndrome, and showed the highest levels of inflammatory biomarkers. Cluster three with predominant proximal LAD stenosis frequently presented with acute coronary syndrome and elevated troponin levels. Cluster four with high-grade stenoses throughout had the oldest patients with the highest overall cardiovascular risk. All-cause and cardiovascular mortality differed significantly between the clusters.

**Conclusions:**

We identified four phenotypic subgroups of CAD bearing distinct demographic and biochemical characteristics with differences in prognosis, which may indicate multiple disease entities currently summarized as CAD.

## Introduction

Coronary artery disease (CAD) is a leading cause of death worldwide (1). While underlying pathophysiological mechanisms of plaque genesis, propagation and rupture have been studied extensively, varying outcomes in individuals with CAD point at underlying pathomechanisms relevant to disease prognosis that still elude our present mechanistic understanding of atherogenesis.

CAD has been recognized as an inflammatory disease of the arterial wall propagated by a large number of cardiovascular risk factors (2). Among these, arterial hypertension, hyperlipidemia, diabetes mellitus, obesity or a history of tobacco consumption are known to effectuate plaque genesis and growth by attracting monocyte-derived cells into the arterial wall and inducing pro-inflammatory processes through chemokine and cytokine secretion. Moreover, additional factors such as chronic kidney disease or chronic inflammatory diseases are linked to CAD progression (3).

Despite extensive knowledge on the systemic risk factors of CAD progression, the vast variety of temporospatial distribution as well as severity of coronary stenoses between or even within individual patients remains inexplicable. Beyond traditional cardiovascular risk factors, numerous hypotheses ascribe a crucial role to factors such as shear stress (4) or inflammatory processes involving the vasa vasorum (5) in atherosclerotic lesion development. Considering the variety of coronary artery disease despite identical risk profile, we hypothesized that specific patterns of coronary atherosclerosis may exist, potentially pointing at different underlying pathophysiological entities.

To test our hypothesis, we performed an unbiased analysis of catheterization reports from the Department of Cardiology at Heidelberg University Hospital. Based on a cluster analysis, we intended to identify specific coronary artery disease patterns. Furthermore, we tested whether the specific patterns could be reproduced and associated to specific cardiovascular risk profiles in an independent cohort (LURIC) and whether these were relevant in terms of overall prognosis.

## Patients and Methods

### Patient Cohorts

#### Heidelberg Coronary Catheterization Cohort

From the database of the Department of Cardiology at University Hospital Heidelberg, consecutive medical records including complete angiographic data of 4,344 patients undergoing coronary angiography between 2014 and 2016 were used for analysis of spatial distribution of coronary stenoses in the hypothesis-generating step of this study. Patients with chronic non-cardiac diseases, a history of malignancy within the past 5 years or those with previous coronary artery bypass graft procedure were excluded. A sample of 495 patients for whom a complete set of clinical and demographical data could be attained from medical records were used for comparative analysis with the LURIC cohort. An overview of the complete data set from the Heidelberg cohort sample is provided as Online Resource 4 (Supplementary Material).

#### Ludwigshafen Risk and Cardiovascular Health Study

The LURIC study consists of 3,129 patients of German descent hospitalized for coronary angiography between 1997 and 2000 at a tertiary care center in Southwestern Germany (6). To limit clinical heterogeneity, individuals suffering from acute illnesses other than acute coronary syndromes were excluded.

#### Structural Comparison Between Heidelberg Complete Cohort and LURIC Study Cohort

Compared to the LURIC cohort, patients in the Heidelberg complete cohort were older on average (67.9 ± 11.5 years vs. 62.4 ± 10.6 years) and less likely to be male [67.3 vs. 73.8% male; Online Resource 4 (Supplementary Material)]. They were more likely to be hypertensive (77.4 vs. 44.5%), more likely to have a history of nicotine abuse (51.2 vs. 19.6%), and more likely to present with an acute coronary syndrome (31.7 vs. 27.1%). Consecutively, patients in the Heidelberg cohort were more likely to show impaired left ventricular ejection fraction (LV-EF <40% in 26.7 vs. 7.5% of patients) as well as lower renal function (mean eGFR 78 ± 25 ml/min vs. 82 ± 20 ml/min). Mean levels of HsTnT (194 ± 1,214 ng/l vs. 87 ± 375 ng/l) and NT-proBNP (2,902 ± 15,693 ng/ml vs. 871 ± 2,049 ng/ml) were higher compared to patients of the LURIC cohort. Conversely, patients in the Heidelberg cohort were less likely to be obese (38.0 vs. 61.8%) and less likely to suffer from hyperlipidemia (63.8 vs. 68.5%) with an overall more favorable LDL/HDL ratio. No significant difference was observed in the prevalence of diabetes mellitus (37.3 vs. 39.4%) and levels of high-sensitive C-reactive protein (mean hs-CRP 7.4 ± 19.9 μg/ml vs. 8.7 ± 18.0 μg/ml).

### Ethics Approval

This study was conducted in accordance with the 1975 Declaration of Helsinki and its later amendments, and analysis of patient data in the Heidelberg cohort was performed following approval by the Ethics Committee of the Faculty of Medicine at University of Heidelberg.

The LURIC study was approved by the Ethics Committee at the “Ärztekammer Rheinland-Pfalz” and was conducted in accordance with the 1975 Declaration of Helsinki and its later amendments.

### Clinical Definitions

Coronary artery disease (CAD) was examined angiographically. Routine angiography comprised four to six projections of the left and two to three projections of right coronary artery. For each coronary artery segment, the highest degree of stenosis seen in any projection was reported. Significant coronary stenosis was defined as greater 50% in at least one of the 15 coronary segments according to the American Heart Association. Assessment of stenoses was done independently by two experienced interventional cardiologists, one of whom was not present during angiography. Consensus between independent estimates regarding degree of stenosis was found to be >90% for all angiographic reports.

Diabetes mellitus was defined according to the American Diabetes Association 2010 as increased fasting (>125 mg/dl) and/or post-challenge (2 h after 75 g oral glucose >200 mg/dl) glucose and/or elevated glycated hemoglobin (>6.4%) and/or history of diabetes mellitus (7). Hypertension was defined as a systolic blood pressure >140 mmHg and/or diastolic blood pressure >90 mmHg or antihypertensive treatment.

### Terminology

The term “coronary artery disease phenotype” in the context of this study is defined as the different patterns of spatial distribution of high-grade stenoses across the coronary branches according to the Gensini scheme identified by two-step cluster analysis. For the purpose of simplicity, these patterns are henceforth referred to as “CAD phenotypes,” “(phenotypic) clusters,” or simply “phenotypes.”

### Statistical Analysis

Statistical analyses were conducted using SPSS version 22.0 (IBM Corps 2013), figures were prepared using Prism (GraphPad 2017). All values are stated as mean ± standard deviation for normally distributed parameters or median with interquartile range for non-normally distributed parameters, respectively, unless otherwise specified. *p* ≤ 0.05 were assumed to be of statistical significance. Descriptive analysis of demographic, laboratory and angiographic data as well as a comparative analysis of female and male patients with respect to all parameters was performed. Normal distribution and deviation thereof were tested for each parameter using the Kolmogorov–Smirnov-Test. Statistical tests included Student's *t*-test for independent variables, non-parametric Mann–Whitney-*U*-Test for non-normally distributed and/or interval-scaled variables or the χ^2^-test for nominally scaled variables.

#### Identification of Clusters

Cluster analysis was performed using the dichotomized stenosis values (>50%) of the 15 coronary segments. To determine the appropriate number of clusters, a random sample of 100 patients was taken for hierarchical agglomerative clustering using the squared Euclidian distance. For stepwise fusion of clusters, Complete Linkage and Ward's method were applied. The number of clusters determined through the Elbow method was used as basis for consecutive *two-step cluster* (*TSC*) *analysis*. TSC was conducted using the dichotomized stenosis values of the 15 coronary segments as categorical cluster variables with the log-likelihood ratio as a measure of distance. In the first step of cluster formation, pre-clusters were formed according to the BIRCH algorithm as described previously (8). In the second step, these pre-clusters were further summarized to form clusters using a hierarchical algorithm. A total of six test runs were performed with varying sequences of patients to ensure maximum consistency within each cluster. All patients recruited from each of the two cohorts were subsequently included in cluster analysis. Furthermore, an analysis of discriminance with formed clusters as categorical variables and dichotomized coronary stenoses as dependent variables were used to determine the likelihood by which an individual patient could be assigned to his/her respective cluster.

#### Characterization of Clusters

Each cluster underwent descriptive analysis to allow for comparison between the individual clusters using univariate analysis of variance (ANOVA) with *post-hoc* Tukey test for normally distributed parameters, non-parametric Kruskal–Wallis test for non-normally distributed parameters and the χ^2^-test, each test using cluster affiliation as a test variable. Associations between cluster affiliation and demographic or laboratory parameters were investigated by ANOVA with *post-hoc* Tukey test in the LURIC cohort. Logistic regression was performed with stepwise inclusion of age, gender, BMI, eGFR, family history, systolic blood pressure, tobacco, diabetes, and LDL. Further, a multivariate Cox regression analysis was performed to determine cardiovascular mortality adjusted for all included demographic and laboratory parameters (age, gender, BMI, diabetes, dyslipidemia, systolic blood pressure, tobacco use, family history, LV function, NT-proBNP, TnT, hs-CRP, and white blood count). Cumulative survival within each cluster was visualized using Kaplan–Meier analysis. Receiver operating characteristic (ROC) analysis was performed using cardiovascular survival as status variable and age <60 years, female sex, no family history for MI, no hypertension or dyslipidemia as well as cluster 1 affiliation as test variables.

## Results

### Atherosclerotic Plaques Are Not Equally Distributed Among Coronary Arteries

**Figure 1:**
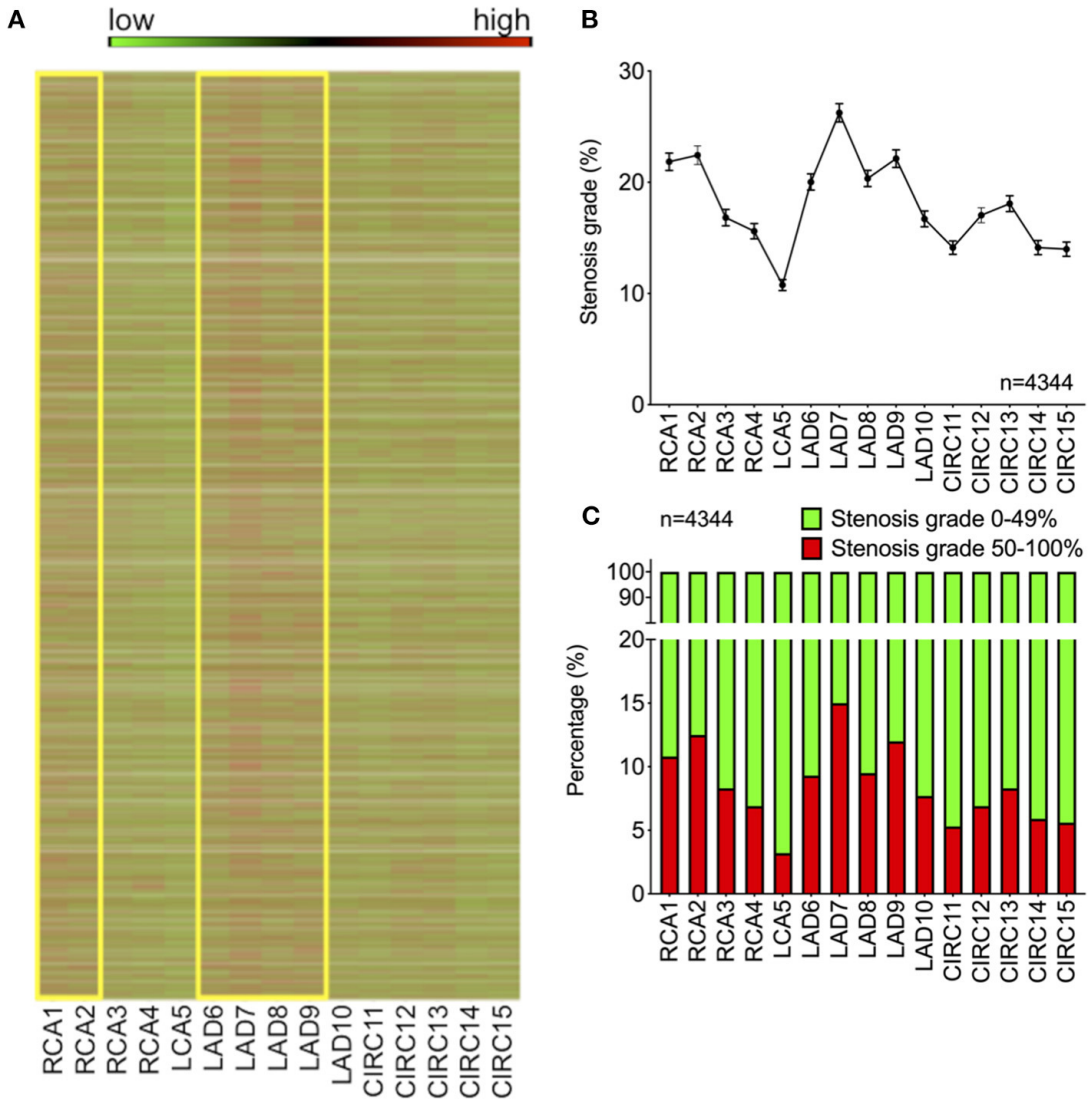
Distribution of plaques among the coronary segments of the Gensini scheme shows specific predilection sites of critical stenosis. **(A)** Heat map color-coded for degree of stenosis for each patient in the cohort (green = 0%, red = 100%). Yellow boxes indicate proximal LAD and RCA regions. **(B)** Average stenosis grade across the 15 Gensini segments (shown as mean ± SEM). **(C)** Percentage of high-grade stenoses >50% (shown in red) across the 15 Gensini segments (LAD, left anterior descending artery; RCA, right coronary artery).

Consecutive coronary angiography reports from the Heidelberg cohort (*n* = 4,344) were analyzed for distribution of coronary stenoses within the 15 coronary segments according to the Gensini scheme. Patients were 65.8 ± 13.2 years of age and 58.4% (2,539) were male. Heat mapping of stenosis >50% of vessel diameter revealed unequal distribution of critical stenoses among the coronary segments (Figure 1A). Visualization furthermore showed an accumulation of high-grade stenoses in the regions of the proximal right (RCA, segments 1 and 2) and left anterior descending artery (LAD, segments 6–9) (Figures 1B,C).

### Coronary Artery Disease Can Be Subdivided Into Four Distinct Phenotypic Clusters

**Figure 2:**
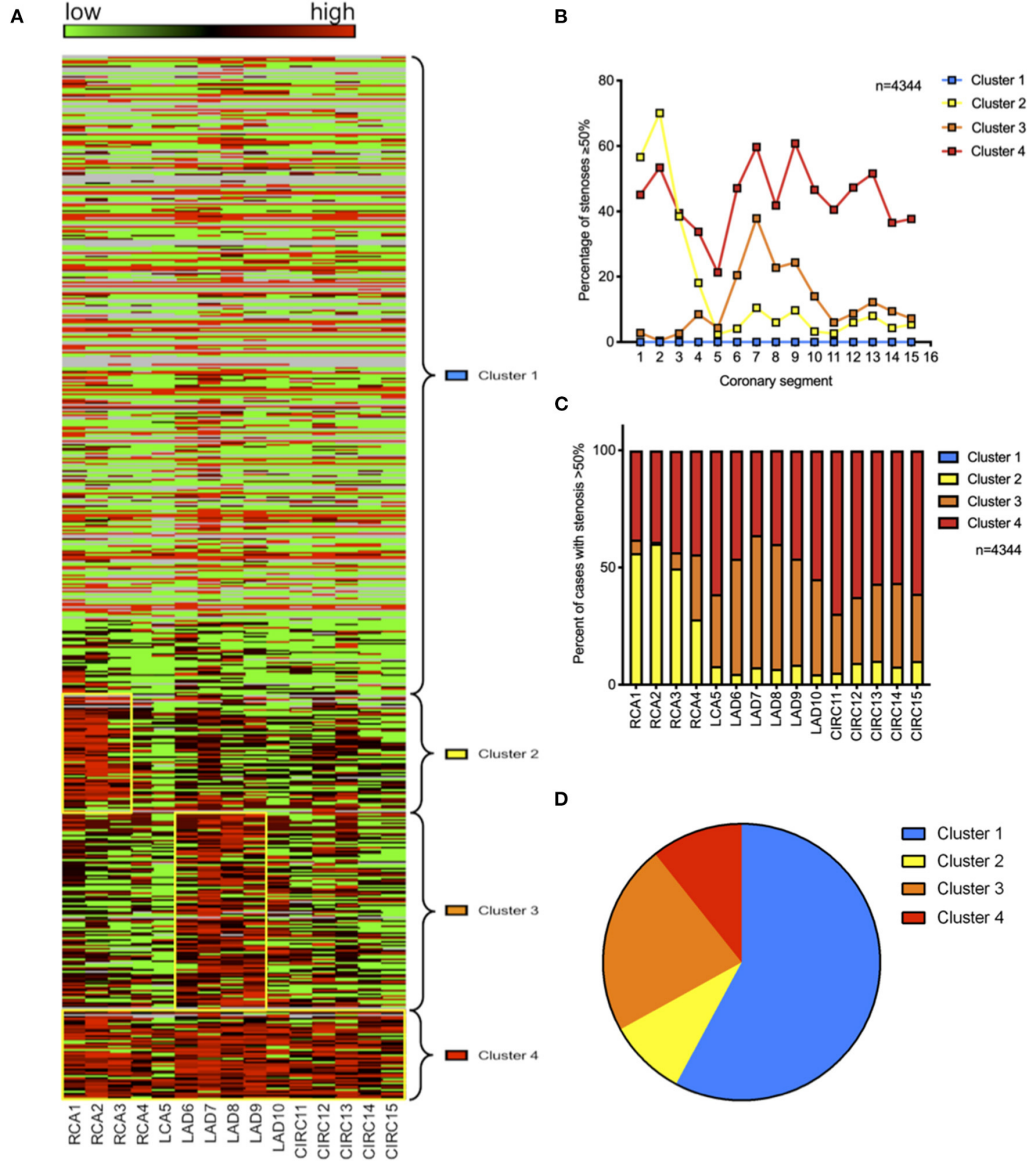
Cluster analysis reveals four distinct phenotypes of coronary artery disease. **(A)** Heat map indicating the degree of stenosis by coronary segment (green = 0%, red = 100%) with free clustering of coronary segments to allow for best fit; yellow boxes indicate high grade stenoses in clusters 2, 3, and 4. **(B)** Average stenosis grade across the 15 Gensini segments within each cluster. **(C)** Percentage of stenoses >50% per coronary segment and cluster of all stenoses >50%. (**D)** Prevalence of the four phenotypic clusters within the cohort.

To determine whether aforementioned findings could be categorized into distinct groups of CAD patients exhibiting a similar CAD phenotype, we performed a cluster analysis of our patient cohort using the 15 Gensini segments as categorical variables. Using Elbow's method, four clusters were found to show the highest cluster quality as measured by the silhouette value of cohesion and separation. A subsequent two-step cluster analysis identified four distinct phenotypic clusters of coronary artery disease. Figure 2A shows the rearrangement of the heat map in Figure 1A according to these four clusters revealing a cluster 1 (blue = “irregularities,” 58%) with predominantly mild vessel wall irregularities devoid of critical stenoses (Figures 2B–D). Cluster 2 [yellow = “RCA,” 9%; OR = 5.41 (3.42–8.56), *p* < 0.001] was characterized by high-grade stenosis of the proximal right coronary artery (segments 1–2), whereas cluster 3 [orange = “LAD,” 22%; OR = 13.31 (7.80–22.74), *p* < 0.001] showed high-grade stenosis of the proximal LAD (segments 7–9). Patients in cluster 4 [red = “diffuse,” 11%; OR for at least one stenosis >50% in all three coronary branches 23.53 (9.72–56.94), *p* < 0.001] exhibited diffuse high-grade stenoses of all three coronary branches (Figures 2B–D). Coronary angiograms representative of each cluster are shown in Online Resource 1 (Supplementary Material).

### CAD Clusters Can Independently Be Reproduced in the LURIC Cohort

As validation of our results, comparative analysis of the spatial distribution of critical stenoses across the 15 Gensini segments in the LURIC cohort revealed similar results to those obtained in the Heidelberg cohort, with prominent peaks in the area of the proximal RCA and the proximal LAD [Online Resource 2A/B/C (Supplementary Material)]. Furthermore, all four clusters could be identified in this cohort, with similar prevalence rates and strong association with high-grade stenosis in their respective coronary branches [cluster 1 (blue = “irregularities,” 50%), cluster 2 [yellow = “RCA,” 22%; OR = 4.64 (3.84–5.60), *p* < 0.001], cluster 3 [orange = “LAD,” 20%; OR = 6.58 (5.25–8.24), *p* < 0.001], cluster 4 [red = “diffuse,” 9%; OR for at least one stenosis >50% in all three coronary branches 35.31 (17.10–72.88), *p* < 0.001]. These results confirm the concept of specific coronary artery disease patterns as a common phenomenon in two independent Central European coronary artery disease cohorts.

### The Four Clusters Exhibit Distinct Demographic and Biochemical Properties

**Figure 3:**
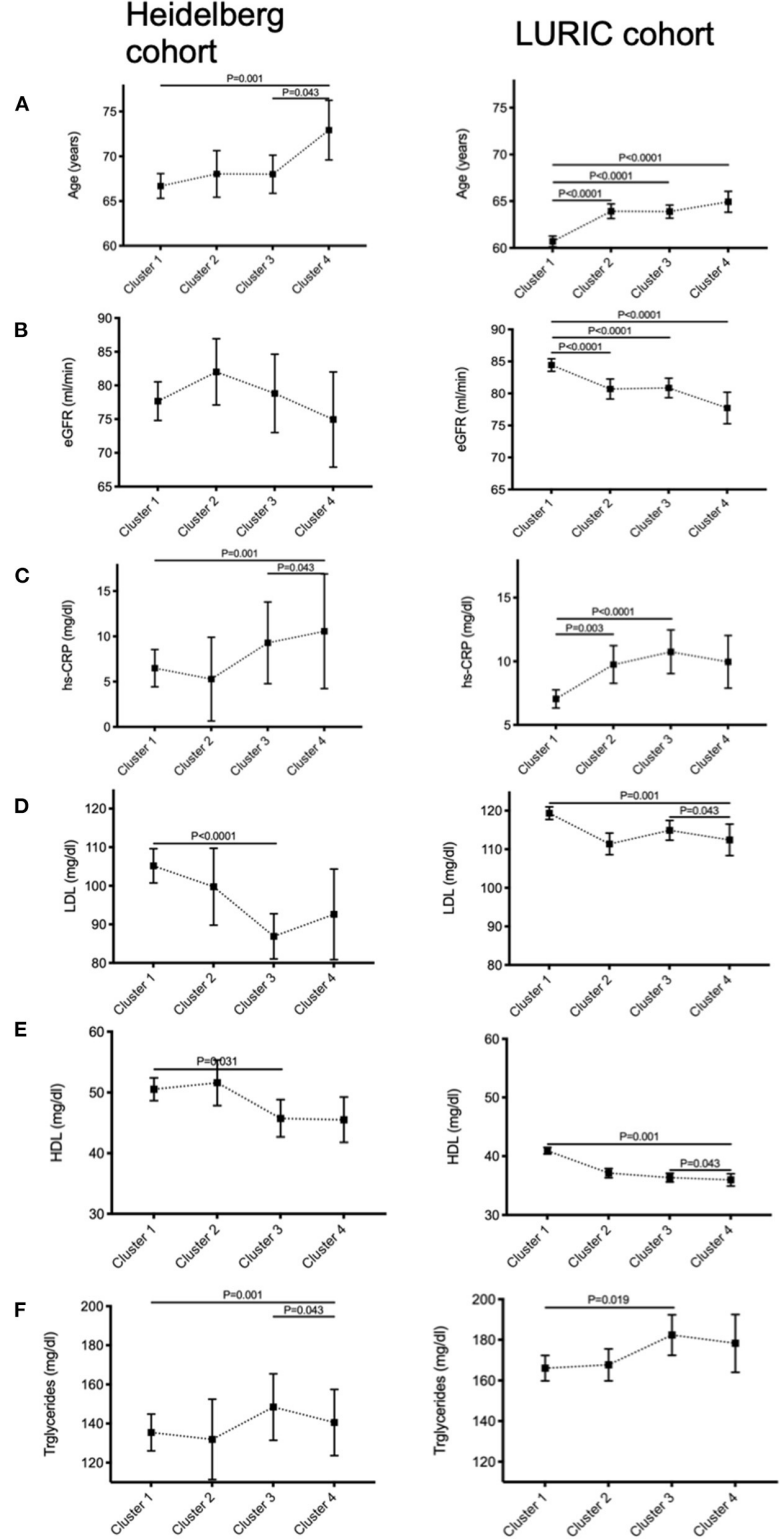
Demographic characterization of each cluster reveals significant differences in cardiovascular risk profile. Comparison between the four phenotypic clusters of the Heidelberg “complete cohort” and the LURIC cohort with respect to **(A)** age, **(B)** renal function, **(C)** residual inflammation quantified by high-sensitive C-reactive protein levels (hs-CRP), **(D)** LDL-, **(E)** HDL-cholesterol, and **(F)** triglyceride levels using analysis of variance (ANOVA).

Comparative analysis of demographic and biochemical data within the individual clusters in the LURIC cohort identified a set of characteristic traits unique to each cluster [Online Resource 3 (Supplementary Material)]. Figure 3 shows ANOVA results characterizing the four clusters. Results of logistic regression to determine specific characteristics of each cluster are shown in Online Resource 5 (Supplementary Material).

In brief, patients in cluster 1 (blue = “irregularities”) were young [HR for age 0.142 (0.088–0.228), *p* < 0.001] and female [HR = 3.367 (2.847–3.981), *p* < 0.001], they display a normal renal function [HR for eGFR 1.464 (1.108–1.933), *p* < 0.01], absence of diabetes [HR for diabetes mellitus 0.620 (0.530–0.725), *p* < 0.001] and low inflammatory activity as seen by low hs-CRP [HR for hs-CRP 0.857 (0.808–0.908), *p* < 0.001] and low LDL levels [HR for LDL 0.410 (0.327–0.516), *p* < 0.001]. Cluster 2 (yellow = “RCA”) had a higher percentage of male individuals [HR = 1.783 (1.486–2.141), *p* < 0.001], patients were older [HR = 4.136 (2.555–6.697), *p* < 0.001], and more likely to be diabetic [HR for diabetes mellitus 1.297 (1.104–1.523), *p* < 0.01]. Cluster 3 (orange = “LAD”) displayed the highest percentage of patients who initially presented with an acute coronary syndrome [HR = 2.208 (1.786–2.732), *p* < 0.001], which is reflected by the highest average high-sensitive troponin T levels of all clusters [HR = 1.357 (1.167–1.579), *p* < 0.001]. Also, hs-CRP levels were higher than in clusters 1 and 2 [HR = 1.206 (1.125–1.292), *p* < 0.001]. Patients in cluster 4 (red = “diffuse”) were the oldest on average [HR = 8.146 (3.465–19.151), *p* < 0.001] and most likely male [HR = 3.378 (2.433–4.695), *p* < 0.001]. Patients in this cluster were found to have the highest overall cardiovascular risk profile including family history [HR = 1.530 (1.199–1.952), *p* = 0.001], high triglycerides, hs-CRP [HR = 1.015 (1.004–1.026), *p* < 0.01] as well as diabetes [HR = 1.390 (1.093–1.768), *p* < 0.01].

### Coronary Artery Disease Phenotype Affects Short-Term Cardiovascular Survival

**Figure 4:**
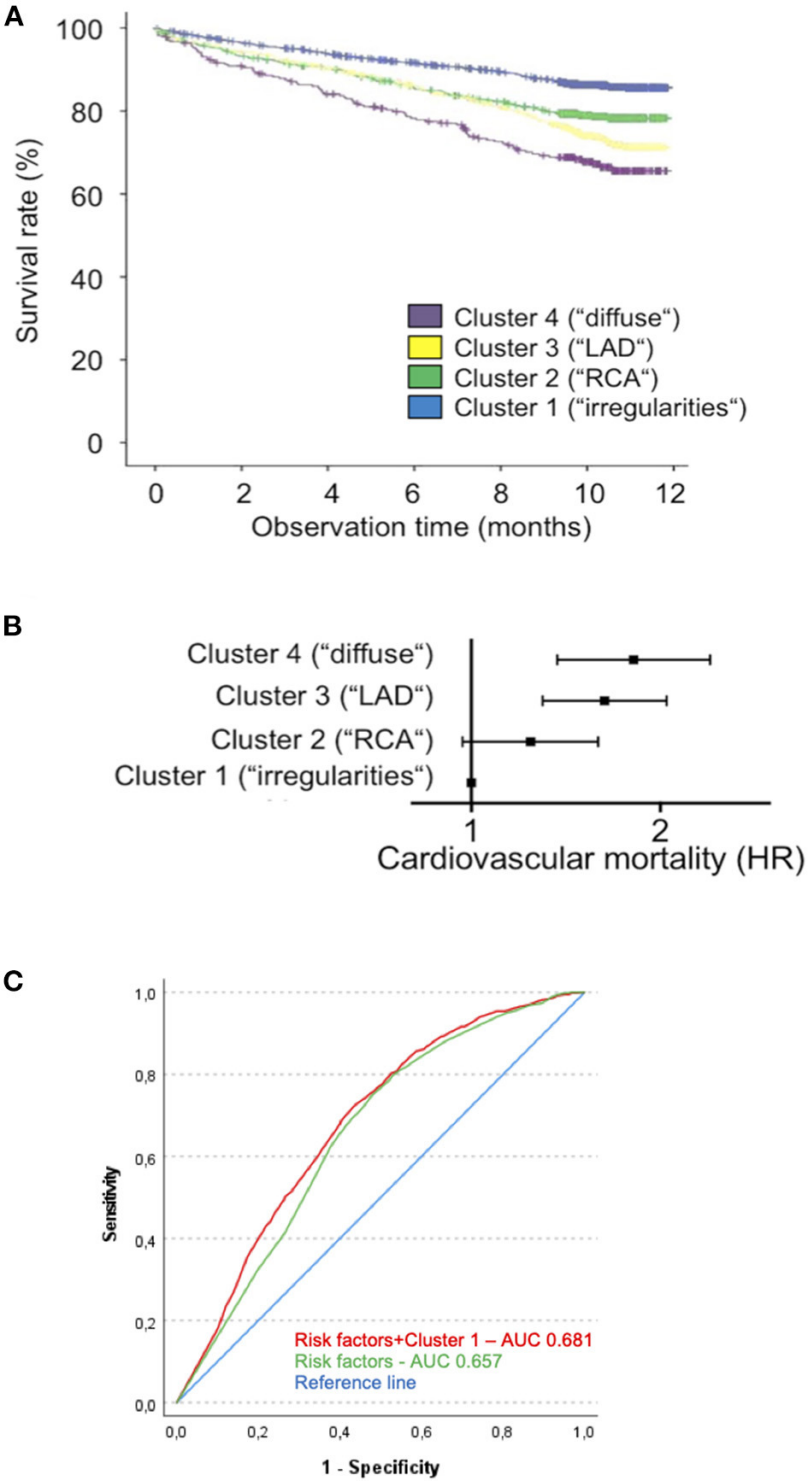
Cluster affiliation is associated with differences in prognosis. **(A)** Kaplan–Meier curve for cardiovascular survival over a 12-month time period for clusters 1 (blue), 2 (green), 3 (yellow), and 4 (purple) in the LURIC cohort. **(B)** Forest plot indicating hazard ratios for cardiovascular death in each cluster (using cluster 1 as reference) as determined by multivariate analysis adjusted for age, gender, BMI, Diabetes, dyslipidemia, systolic blood pressure, tobacco use, family history, LV function, NT-proBNP, hs-TnT, hs-CRP, and white blood count. Cluster 2: HR = 1.242, 0.986–1.592, n.s.; cluster 3: HR = 1.630, 1.298–2.047, *P* < 0.0001; cluster 4: HR = 1.814, 1.371–2.400, *P* < 0.0001. **(C)** ROC curve demonstrating predictive ability of a favorable cardiovascular risk profile (age < 60 years, female sex, no family history for MI, no hypertension or dyslipidemia) alone or in combination with cluster 1 affiliation with regard to cardiovascular survival (BMI, body mass index; NT-proBNP, n-terminal pro-brain natriuretic protein; hs-TnT, high-sensitive troponin T; hs-CRP, high-sensitive C-reactive protein).

To investigate whether CAD phenotype holds a prognostic value in terms of cardiovascular mortality, Kaplan–Meier analyses were performed in the LURIC cohort (Figure 4A). Even after adjustment for age, gender, body mass index, diabetes mellitus, dyslipidemia, hypertension, tobacco use, family history, LV-function, NT-proBNP, hs-TnT, and hs-CRP levels, CAD phenotype significantly predicted cardiovascular mortality [Figure 4B; Online Resource 6 (Supplementary Material)]. Cluster 1 with only mild CAD showed the highest survival, whereas patients in cluster 4 with diffuse high-grade coronary stenoses had the poorest overall survival over the 12-month observation period (median survival 8.9–9.9 years), with a significant increase in mortality both before and after multivariable adjustment [HR unadjusted 2.710 (2.107–3.485), *p* < 0.001; HR adjusted 1.784 (1.350–2.358), *p* < 0.001]. Of note, patients in cluster 3 with prominent stenosis of the left anterior descending (LAD) artery had a poorer 12-month survival [HR unadjusted 2.204 (1.653–2.478), *p* < 0.001; HR adjusted 1.607 (1.282–2.015), *p* < 0.001] compared to those of cluster 2 with predominantly high-grade stenosis of the proximal to mid right coronary artery (RCA) [HR unadjusted 1.620 (1.297–2.025), *p* < 0.001; HR adjusted 1.225 (0.956–1.569, *p* = 0.09)].

Likewise, receiver operating characteristic (ROC) analysis of survival prognosis using either a favorable cardiovascular risk profile alone (age <60 years, female sex, no family history for MI, no hypertension or dyslipidemia) or in combination with cluster 1 affiliation in the LURIC cohort showed a significant increase in the area under the ROC curve [ROC AUC = 0.681 (0.659–0.703), *p* < 0.001 vs. 0.657 (0.635–0.679), *p* < 0.0001; difference in AUC = 0.024 (0.012–0.037), *p* < 0.001], indicating an additional benefit of cluster 1 affiliation for survival prediction (Figure 4C).

## Discussion

The molecular mechanisms underlying atherogenesis have been extensively studied over the past decades. Despite our knowledge how the systemic risk factors affect CAD progression, the fact that coronary atherosclerosis can differ significantly between patients with similar risk profile remains enigmatic. Moreover, it is anything but clear why even in a single individual atherosclerotic plaque burden may substantially differ between coronary segments. The explanation that this heterogeneity is entirely due to shear stress remains unsatisfactory.

Our attempt to shed light on this obscurity is an unbiased approach analyzing lesion distribution throughout the coronary artery tree in a cohort of >4,000 consecutive patients at Heidelberg University Hospital. We thereby—for the first time—reveal four distinct patterns of coronary atherosclerotic plaque distribution, which could subsequently be independently confirmed in >3,000 patients of the LURIC study. In the LURIC cohort, these clusters correlate with specific demographic and clinical parameters, and are furthermore associated independently with cardiovascular mortality. Patients in cluster 1 (“irregularities”) with a relatively young age and low cardiovascular risk exhibiting diffuse vessel wall irregularities devoid of critical stenoses may represent a form of “baseline CAD” expressed in every individual with increasing age, the extent of which may be modified as the consequence of cardiovascular risk factors. In this respect, the CAD phenotype seen in cluster 1 may represent the incipient plaque formation in coronary arteries of children and young adults observed in a previous study (9). By contrast, patients belonging to cluster 2 (“RCA”) showed signs of an elevated metabolic risk, associated with a CAD phenotype predominantly affecting the proximal right coronary artery. While the pathomechanisms resulting in this particular and prognostically relevant distribution of coronary atherosclerosis are unclear, a possible link may be the marked presence of chronic low-grade inflammation present in individuals with metabolic syndrome (10) that was observed in our cohort. An elevated inflammatory response was found to be a prominent hallmark of both clusters 3 (“LAD”) and 4 (“diffuse”), the latter being associated with the most unfavorable cardiovascular risk profile of all clusters as well as a strong family history of CAD, resulting in diffuse high-grade stenoses of all coronary branches with the poorest short-term survival. Notably, patients in cluster 3 who most often presented with an acute coronary syndrome and were found to exhibit critical stenosis of the proximal left anterior descending artery may point toward a propensity of unstable plaque formation in these coronary segments. This is in line with proximal LAD stenosis being a prognostically relevant target for coronary intervention or bypass surgery (11).

Analogous to myocardial infarction, previously believed to be one homogenous entity with linear pathophysiology that has now been recognized as the common pathway of a number of pathologies reaching from superficial plaque erosion to plaque rupture (12), CAD itself may similarly be the end product of a number of pathophysiologically distinct disease mechanisms. In our study, we are first to associate commonly known risk factors of cardiovascular disease such as age, male gender or the metabolic syndrome as well as emerging risk factors such as systemic inflammation (13) with a specific spatial pattern of coronary artery disease, supporting this hypothesis of differential pathomechanisms in coronary atherosclerosis with potential prognostic as well as therapeutic value. Previous work such as the APPROACH trial has provided extensive evidence on the impact of coronary plaque burden on long-term survival (14), and few assumptions on the relevance of location of critical stenosis for overall survival have been made previously (15). Overall, however, current knowledge on an association of spatial distribution of CAD with specific cardiovascular risk factors is scarce.

Limitations to our study are grounded on the mode of data collection and availability of data in our study cohort. The degree of stenosis for each segment of the Gensini scheme is usually documented based on the interventionalist's estimate and is therefore prone to some degree of variation due to inter-observer bias (16). However, each angiography was analyzed independently by two experienced interventional cardiologists, one of which was not present during the procedure. Consensus respective to degree of stenosis was >90% between independent reports for all angiographies. Furthermore, our data do not take into account variation of coronary anatomy (i.e., dominant left or right coronary artery, coronary anomalies). However, based on the large number of catheterization reports, this bias should not significantly affect our overall results.

Moreover, we cannot exclude that our findings are at least partly affected by a temporospatial variation of disease progression, although multivariate analysis including patient age as confounder still demonstrates significant association between coronary phenotype and cardiovascular mortality. Nevertheless, as there is no defined incipient point of coronary atherogenesis, and development of coronary atherosclerotic plaques may have started at an earlier age in some individuals, we cannot fully exclude the possibility of some form of temporal variation as a confounding factor.

Furthermore, the lack of intravascular imaging and functional data may affect the quality of analysis, since particularly in intermediate-degree stenoses, further diagnostic assessment of functional relevance of a coronary stenosis is indicated, thus potentially guiding the interventionalist's decision in favor of percutaneous coronary intervention. This additional diagnostic information may help improve the quality of dichotomization of coronary stenoses in our cluster analysis. On that note, evolution of diagnostic standards and non-invasive imaging along with a sharp improvement in the sensitivity of cardiac troponin assays in the last decades may implicate a certain degree of variance in the indication for angiographic testing between the two cohorts, the LURIC cohort recruiting patients as early as 1997. Albeit a recent diagnostic shift away from invasive angiography and toward increased non-invasive imaging as a confounding factor in this study, all patients included herein underwent guideline-conform diagnostic steps, and all troponin tests used in our study cohorts were performed using state-of-the-art high sensitivity assays. Lastly, due to the predominantly Caucasian cohorts used for this analysis, genetic heterogeneity may pose a confounding factor in the translation of the results obtained herein to an ethnically different or more diverse population.

In conclusion, our study sets a precedent in identifying four specific coronary artery disease phenotypes, which are correlated with a specific cardiovascular risk profile, thus pointing at the possible existence of different disease entities within the pathology presently known as coronary artery disease. These different CAD entities may reflect different underlying pathomechanisms dictating spatial distribution of atherosclerotic lesion formation beyond the current holistic assumption that plaque location is largely dependent on hemodynamics (17) and otherwise somewhat arbitrary. Furthermore, the evidence provided herein may indicate a difference in disease prognosis dependent on CAD phenotype. Further studies are needed to investigate the existence of distinct pathomechanisms associated with the phenotypes identified herein that may advance our understanding of the pathophysiological nuances of coronary artery disease, potentially providing novel targets for individualized preventative therapies.

## Data Availability Statement

The raw data supporting the conclusions of this article will be made available by the authors upon reasonable request, without undue reservation.

## Ethics Statement

The studies involving human participants were reviewed and approved by Ethics Committee at the Faculty of Medicine at University of Heidelberg. The patients/participants provided their written informed consent to participate in this study. The LURIC study was approved by the Ethics Committee at the “Ärztekammer Rheinland-Pfalz” and was conducted in accordance with the 1975 Declaration of Helsinki and its later amendments.

## Author Contributions

JK, YN, and CG postulated the hypothesis and designed the framework of this study. YN and JK did the majority of data collection and processing. JK and CG were in charge of drafting the manuscript. WM and HK held advisory functions throughout the conceptualization and realization of this study. WM contributed to cluster analysis using the LURIC database. MA, CE, GaD, FL, MK, and GrD contributed to data collection and processing. All authors contributed to the article and approved the submitted version.

## Funding

This study was funded by the Faculty of Medicine at Heidelberg University. Partial financial support was received from the Cardiology Career Program (CCP) of the University Hospital Heidelberg (to YN).

## Conflict of Interest

JK, YN, and CG: pending patent, CoroScore—ein automatisiert bestimmbarer Angiographie-basierter Score zur Verbesserung der kardiovaskulären Risikoprädiktion. MK: personal fees from Bayer. WM: grants from Siemens Healthineers, Aegerion Pharmaceuticals, AMGEN, AstraZeneca, Sanofi, Alexion Pharmaceuticals, BASF, Abbott Diagnostics, Numares AG, Berlin-Chemie, Akzea Therapeutics, Bayer Vital, bestbion dx, Boehringer Ingelheim, Immundiagnostik, Merck Chemicals, MSD Sharp and Dohme, Novartis, Olink Proteomics. Personal fees from Aegerion Pharmaceuticals, AMGEN, Sanofi, Alexion Pharmaceuticals, BASF, Abbott Diagnostics, Numares AG, Berlin-Chemie, Akzea Therapeutics. HK: personal fees from AstraZeneca, Bayer Vital, Daiichi Sankyo, Boehringer Ingelheim, Roche Diagnostics. WM is employed by Synlab GmbH. The remaining authors declare that the research was conducted in the absence of any commercial or financial relationships that could be construed as a potential conflict of interest.

## Publisher's Note

All claims expressed in this article are solely those of the authors and do not necessarily represent those of their affiliated organizations, or those of the publisher, the editors and the reviewers. Any product that may be evaluated in this article, or claim that may be made by its manufacturer, is not guaranteed or endorsed by the publisher.
