# Supplementary material for: Identification of Specific Coronary Artery Disease Phenotypes Implicating Differential Pathophysiologies
Source: Front Cardiovasc Med. 2022 Mar 10;9:778206. doi: 10.3389/fcvm.2022.778206 (PMC8960070; doi:10.3389/fcvm.2022.778206)
Supplement: Supplementary file 1 [file Data_Sheet_1.pdf]

Supplementary Information

Identification of specific coronary artery disease phenotypes implicating differential pathophysiologies

Jona B. Krohn[†], Y Nhi Nguyen[†], Mohammadreza Akhavanpoor, Christian Erbel, Gabriele Domschke, Fabian Linden, Marcus E. Kleber, Graciela Delgado, Winfried März, Hugo A. Katus, Christian A. Gleissner*

*Correspondence:
Prof. Dr. med. Christian A. Gleissner, gleissner.christian@rottalinnkliniken.de

Online Resource 1: Representative coronary angiograms of patients from the Heidelberg cohort for clusters 1 through 4 (A-D).

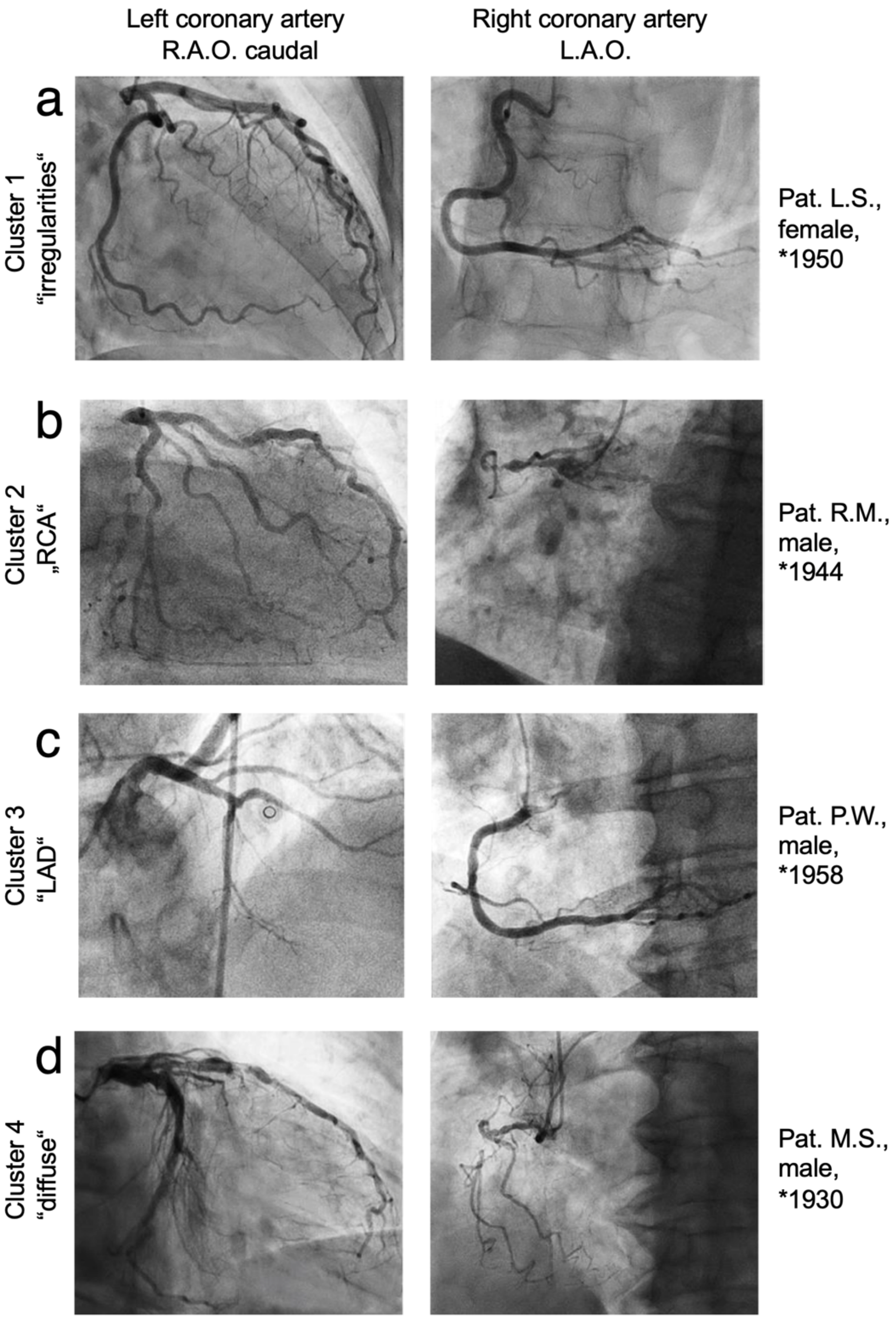

Online Resource 2: The distinct CAD clusters can be reproduced in the independent LURIC cohort. (A) Average stenosis grade and **(B)** percentage of high-grade stenoses >50% (shown in red) across the 15 Gensini segments. **C.** Prevalence of the four phenotypic clusters within the LURIC registry.

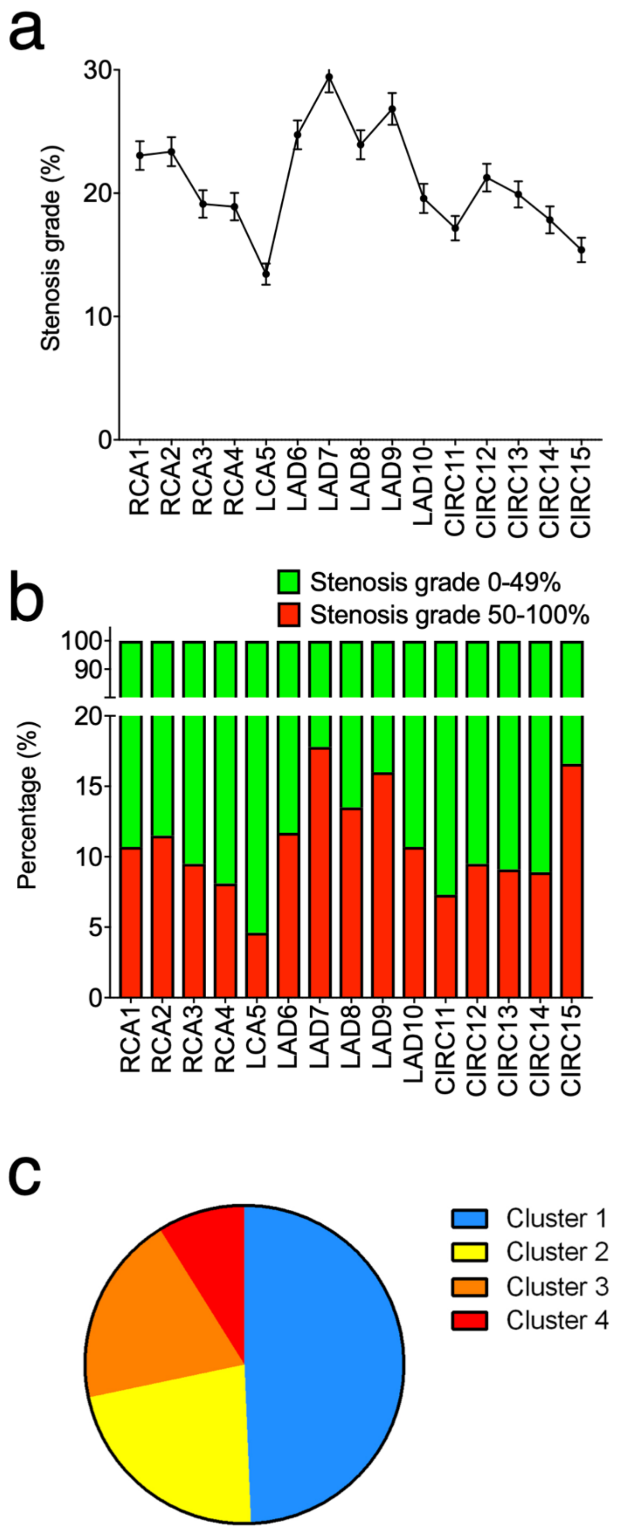

Online Resource 3: Clinical and demographic data of LURIC patients. Percentage of total displayed in parentheses ().

	All patients (n=3129)	Cluster 1 (n=1543)	Cluster 2 (n=699)	Cluster 3 (n=609)	Cluster 4 (n=278)	P value
**Demographics**						
Age (years)	62.4±10.6	60.7±11.4	63.9±9.4	63.9±9.7	64.9±9.4	<0.001
Gender (male)	2310 (73.8%)	972 (63.0%)	602 (86.1%)	487 (80.0%)	249 (89.6%)	<0.0001
**Clinical parameters**						
Arterial hypertension	1388 (44.5%)	485 (31.4%)	439 (62.8%)	279 (45.8%)	185 (66.5%)	<0.0001
Diabetes mellitus	1230 (39.4%)	497 (32.2%)	313 (44.8%)	279 (45.8%)	141 (50.7%)	<0.0001
Hyperlipidemia	2144 (68.5%)	951 (61.6%)	540 (77.3%)	435 (71.4%)	218 (78.4%)	<0.0001
History of nicotine abuse	611 (19.6%)	320 (20.7%)	159 (22.7%)	90 (14.8%)	42 (15.1%)	<0.0001
Family history	1094 (35.1%)	485 (31.4%)	265 (37.9%)	235 (38.6%)	109 (39.2%)	0.001
Left ventricular ejection fraction (%)	52±7	52±8	53±7	52±7	54±7	<0.0001
Acute coronary syndrome	849 (27.1%)	206 (13.4%)	279 (39.9%)	253 (41.5%)	111 (39.9%)	<0.001
**Laboratory parameters**						
Estimated glomerular filtration rate (ml/min)	82±20	84±20	81±20	81±20	78±20	<0.0001
High sensitivity C-reactive protein (µg/ml)	8.7±18.0	7.0±14.3	10.7±23.1	9.7±18.6	10.0±17.5	<0.0001
High sensitivity troponin T (ng/l)	87±375	42±236	132±482	146±524	90±262	<0.0001
NT-proBNP (ng/ml)	871±2049	700±1511	912±1869	1097±2893	1226±2672	<0.0001
LDL (mg/dl)	116±34	119±33	115±34	111±35	112±35	<0.0001
HDL (mg/dl)	39±11	41±11	36±10	37±10	36±9	<0.0001
Triglycerides (mg/dl)	171±123	166±125	182±135	168±99	178±123	0.019

Online Resource 4: Clinical and demographic data of 495 patients from the Heidelberg cohort. All patients from the Heidelberg cohort (n=495) from which a complete set of clinical/demographic data analogous to the LURIC cohort was available are listed. Interquartile range displayed in brackets [], percentage of total in parentheses (). *ns=not significant*

	All patients (n=495)	multiple comparisons with LURIC	Cluster 1 (n=251)	Cluster 2 (n=77)	Cluster 3 (n=112)	Cluster 4 (n=55)
**Demographics**						
Age (years)	68 [60-76]	older (P<0.001)	68 [59-76]	68 [61-78]	67 [60-77]	74 [65-82]
Gender (male)	333 (67.3%)	more females (P<0.01)	147 (58.6%)	55 (71.4%)	88 (78.6%)	43 (78.2%)
**Clinical parameters**						
Arterial hypertension	383 (77.4%)	more hypertension (P<0.001)	188 (74.9%)	63 (88.1%)	82 (73.2%)	50 (90.9%)
Diabetes mellitus	185 (37.3%)	ns	87 (34.7%)	32 (41.6%)	46 (41.1%)	20 (36.3%)
Hyperlipidemia	316 (63.8%)	less hyperlipidemia (P=0.04)	146 (58.2%)	54 (70.1%)	79 (70.5%)	37 (67.3%)
Obesity	188 (38.0%)	less obese (P<0.001)	97 (38.6%)	26 (33.8%)	46 (41.1%)	19 (34.5%)
History of nicotine abuse	253 (51.2%)	more smokers (P<0.001)	122 (48.6%)	41 (53.3%)	59 (52.7%)	31 (56.4%)
Family history	190 (38.4%)	ns	96 (38.2%)	36 (46.8%)	41 (36.6%)	17 (30.9%)
Left ventricular ejection fraction <40%	132 (26.7%)	lower LVEF (P<0.001)	64 (25.5%)	21 (27.3%)	28 (25.0%)	19 (34.0%)
Acute coronary syndrome	157 (31.7%)	more ACS (P=0.03)	69 (27.5%)	30 (39.0%)	33 (29.5%)	25 (45.5%)
**Laboratory parameters**						
Estimated glomerular filtration rate (ml/min)	80.6 [61.8-96.2]	lower eGFR (P<0.001)	79.6 [60.5-99.5]	86.1 [65.9-97.6]	80.4 [59.9-95.7]	75.7 [54.4-92.0]
High sensitivity C-reactive protein (µg/ml)	2.3 [<2.0-6.7]	ns	2.2 [<2-6.7]	<2.0 [<2.0-3.2]	2.6 [<2.0-7.4]	3.0 [<2.0-8.3]
High sensitivity troponin T (ng/l)	14 [8-31]	higher average hsTnT (P<0.001)	13 [8-22]	14 [8-40]	15 [8-36]	19 [9-78]
NT-proBNP (ng/l)	354 [98-1,231]	higher average BNP (P<0.001)	423 [118-1,331]	149 [61-481]	281 [96-748]	413 [131-2,603]
LDL (mg/dl)	93 [74-119]	lower LDL (P<0.001)	101 [78-127]	89 [75-112]	81 [69-99]	88 [69-102]
HDL (mg/dl)	46 [38-57]	higher HDL (P<0.001)	48 [40-58]	47 [41-62]	42 [35-53]	46 [34-52]

Online Resource 5: Results of logistic regression analysis for each cluster. The following parameters were included stepwise in order to identify those factors that independently associated with each cluster: Age, gender, body mass index, eGFR, family history, systolic blood pressure, tobacco, diabetes, hs-CRP, LDL (*eGFR estimated glomerular filtration rate, hs-CRP high sensitivity C-reactive protein)*

Include stepwise

Cluster	Parameter	HR (95%-CI)	P value	Summary → text
1	Age	0.142 (0.088-0.228)	<0.0001	Young
	Gender	3.367 (2.847-3.981)	<0.0001	Female
	Body mass index	2.076 (1.231-3.499)	0.006	
	eGFR	1.464 (1.108-1.933)	0.007	Normal renal fx
	Family history	0.580 (0.495-0.679)	<0.0001	No family hx
	Diabetes mellitus	0.620 (0.530-0.725)	<0.0001	No diabetes
	hs-CRP	0.857 (0.808-0.908)	<0.0001	Low inflamm.
	LDL	0.410 (0.327-0.516)	<0.0001	Low LDL
2	Age	4.136 (2.555-6.697)	<0.0001	Old
	Gender	0.561 (0.467-0.673)	<0.0001	Male
	Family history	1.179 (1.000-1.391)	0.050	
	Diabetes mellitus	1.297 (1.104-1.523)	0.002	Diabetes
	LDL	1.457 (1.146-1.854)	0.002	High LDL
3	Gender	0.590 (0.475-0.734)	<0.0001	Male
	Family history	1.392 (1.152-1.683)	0.001	Pos. fam. Hx
	Systolic blood pressure	2.208 (1.288-3.784)	0.004	HTN
	hs-CRP	1.206 (1.125-1.292)	<0.0001	Inflammation
	LDL	1.641 (1.235-2.180)	0.001	High LDL
4	Age	8.146 (3.465-19.151)	<0.0001	Very old
	Gender	0.296 (0.213-0.411)	<0.0001	“very” male
	eGFR	0.641 (0.435-0.946)	0.025	bad renal fct
	Family history	1.530 (1.199-1.952)	0.001	strong fam hx
	Diabetes mellitus	1.390 (1.093-1.768)	0.007	strong Diabetes
	LDL	2.012 (1.388-2.916)	<0.0001	Very high LDL

Online Resource 6: Cox regression analysis of cardiovascular mortality associated with CAD phenotype. Analysis was adjusted for age, gender, body mass index, diabetes mellitus, hypertension, dyslipidemia, tobacco use, positive family history for MI, angiographic determination of LV-function, NT-proBNP, hsTnT and hs-CRP. *hs-CRP = high sensitivity C-reactive protein, hs-TnT = high-sensitive cardiac troponin T, NT-proBNP = N-terminal pro-brain natriuretic peptide.*

Parameter	HR (95%-CI)	P value
Age	1.045 (1.032-1.058)	<0.001
Gender	0.903 (0.718-1.135)	0.381
Body mass index	1.007 (0.983-1.032)	0.553
Diabetes mellitus	1.754 (1.459-2.109)	<0.001
Hypertension	1.002 (0.998-1.006)	0.420
Dyslipidemia	0.964 (0.785-1.183)	0.723
Tobacco use	1.162 (0.908-1.488)	0.233
Family history	0.885 (0.727-1.078)	0.224
LV-Function	1.317 (1.139-1.347)	<0.001
NT-proBNP	1.381 (1.263-1.510)	<0.001
hs-TnT	1.015 (0.943-1.093)	0.690
hs-CRP	1.009 (0.970-1.050)	0.651
**CAD phenotype**	**1.238 (1.139-1.347)**	**<0.001**
